# Molybdenum-Modified Titanium Dioxide Nanotube Arrays as an Efficient Electrode for the Electroreduction of Nitrate to Ammonia

**DOI:** 10.3390/molecules29122782

**Published:** 2024-06-11

**Authors:** Huixi Chen, Wenqi Hu, Tingting Ma, Yixuan Pu, Senhao Wang, Yuan Wang, Shaojun Yuan

**Affiliations:** Low-Carbon Technology & Chemical Reaction Engineering Labaratory, College of Chemical Engineering, Sichuan University, Chengdu 610065, China; 18780249858@163.com (H.C.); 13551009872@163.com (W.H.); 13594084176@163.com (T.M.); yixuanpu@icloud.com (Y.P.); senhaow@163.com (S.W.)

**Keywords:** electrochemical nitrate reduction, NH_3_, MoO_x_, nanotube arrays, electrodeposition

## Abstract

Electrochemical nitrate reduction (NO_3_^−^RR) has been recognized as a promising strategy for sustainable ammonia (NH_3_) production due to its environmental friendliness and economical nature. However, the NO_3_^−^RR reaction involves an eight-electron coupled proton transfer process with many by-products and low Faraday efficiency. In this work, a molybdenum oxide (MoO_x_)-decorated titanium dioxide nanotube on Ti foil (Mo/TiO_2_) was prepared by means of an electrodeposition and calcination process. The structure of MoO_x_ can be controlled by regulating the concentration of molybdate during the electrodeposition process, which can further influence the electron transfer from Ti to Mo atoms, and enhance the binding energy of intermediate species in NO_3_^−^RR. The optimized Mo/TiO_2_-M with more Mo(IV) sites exhibited a better activity for NO_3_^−^RR. The Mo/TiO_2_-M electrode delivered a NH_3_ yield of 5.18 mg h^−1^ cm^−2^ at −1.7 V vs. Ag/AgCl, and exhibited a Faraday efficiency of 88.05% at −1.4 V vs. Ag/AgCl. In addition, the cycling test demonstrated that the Mo/TiO_2_-M electrode possessed a good stability. This work not only provides an attractive electrode material, but also offers new insights into the rational design of catalysts for NO_3_^−^RR.

## 1. Introduction

Ammonia (NH_3_) is essential in various industrial sectors, such as chemical production, refrigeration, and pharmaceuticals [1,2]. It also serves as a carbon-free fuel with a high hydrogen density (the NH_3_ molecule has a H content of 17.75% by weight), making it easily storable and transportable through existing infrastructures [3]. The Haber–Bosch process for NH_3_ production stands as the most significant inventions in chemical engineering. It relies on high-temperature (400–600 °C) and high-pressure (200–350 atm) reactions between N_2_ and H_2_ with a suitable catalyst [4,5,6]. Apart from the energy required for heating and pumping, the high-purity H_2_ utilized in this process is predominantly produced from natural gas, resulting in substantial greenhouse gas emissions [7,8]. Hence, there is considerable importance in developing a sustainable and environmentally friendly strategy for NH_3_ production. Electrocatalytic approaches are recognized for their energy-saving and environmentally benign nature, distinguished by simple equipment, mild state, high efficiency, and immense potential for application on a large scale [9,10].

Among these approaches, NH_3_ production from N_2_ and H_2_O has garnered significant attention [11,12]. However, the N≡N bond energy is notably high at 941 kJ mol^−1^, and N_2_ only dissolves sparingly in water [13]. Consequently, the energy utilization of nitrogen reduction reactions in the aqueous environment is limited, with NH_3_ production yields up to three orders of magnitude lower than those of the Haber–Bosch process [14]. Considering this, researchers have explored other nitrogen-containing compounds as NH_3_ synthesis sources and identified NO_3_^−^ as a promising candidate. NO_3_^−^ exhibits good solubility in water, and the N=O bond energy is relatively low at 204 kJ mol^−1^ [15,16]. In addition, NO_3_^−^ is also a significant pollutant due to human activities, including excessive nitrogen fertilizer usage, fossil fuel combustion, and wastewater discharge, contributing to increased nitrate levels in water [17]. The World Health Organization (WHO) recommends that the level of nitrates in drinking water not exceed 50 mg L^−1^ [18]. Once nitrates are ingested by the human body, they undergo metabolism in the digestive system where they are converted into nitrites, thereby presenting a potential carcinogenic hazard [19]. Therefore, utilizing NO_3_^−^ as a nitrogen source for NH_3_ synthesis not only enhances energy utilization efficiency and reduces greenhouse gas emissions, but also addresses the issue of NO_3_^−^ pollution in the environment.

Recently, the electrocatalytic nitrate synthesis of ammonia (NO_3_^−^RR) has been investigated using metals such as Cu, Fe, Pt, Ag, and Mo [20,21,22,23,24]. Among them, Mo has attracted much attention due to its low price and good catalytic performance. Moreover, the nitrate reduction reaction process in nature is facilitated by an enzymatic cascade, where the Mo(IV) cofactor of nitrate reductase catalyzes the conversion of NO_3_^−^ to NO_2_^−^ [25]. This step plays a crucial role in determining the overall reaction rate [26]. Wang et al. reported MoO_2_ nanoparticles supported by molybdenum plate as an electrode for NO_3_^−^RR. Such MoO_2_ nanoparticles delivered a good activity for the conversion of NO_3_^−^ to NH_3_ [27]. Zhong et al. reported a MoO/C catalyst capable of a stable reaction for 50 h, suggesting its potential in the treatment of nitrate-containing wastewater [28]. Song et al. found that MoO_x_ exhibits a high affinity for adsorbing NO_3_^−^ ions [29]. The Mo(IV) site can hinder the adsorption of NO_2_^−^ and contributes to the conversion of *NO to *NOH [29]. Thus, MoO_x_ with a Mo(IV) site can accelerate the conversion of electrochemical NO_3_^−^ to NH_3_ and limit NO_2_^−^ generation, resulting in the efficient production of NH_3_.

Generally, the nanostructure is favorable to enlarge the interface between electrode and electrolyte, and the construction of a nanocomposite can endow the catalyst with more active sites [30,31,32]. Xiong et al. prepared platinum nanoparticles embedded on nickel oxide nanosheets, which can serve as a electrocatalyst for boosting NO_3_^−^ transfer [33]. Wang et al. constructed ultra-small iron oxide nanoparticles on carbon nanotubes, which can enlarge the active surface area and accelerate ion transfer [34]. All these studies have demonstrated that nanomaterials have the effect of promoting full solid-liquid phase contact. As a nontoxic and abundant material, TiO_2_ is one of the hot spots in photochemistry and electrochemistry. TiO_2_ nanotube arrays (TNTAs) can be easily prepared and serve as the substrate with a large surface area to support active materials [35,36,37]. Inspired by this, we anticipated that decorating MoO_x_ onto the surface of TNTAs to form a nanocomposite structure can enlarge the active area and enhance the ion transfer from electrolyte to electrode.

In this work, MoO_x_-loaded TNTAs was prepared on Ti foil (Mo/TiO_2_) by means of an electrodeposition and calcination process. This can directly serve as the electrode for NO_3_^−^RR. The TNTAs as the substrate can offer a large surface area to support MoO_x_. The Mo/TiO_2_ nanocomposite structure was confirmed by SEM and TEM measurement. Notably, XPS measurement further revealed the electron transfer behavior between Ti and Mo atoms. The electron transfer can be modulated by regulating the concentration of molybdate during the electrodeposition process. By optimizing the electrodeposition conditions, the obtained Mo/TiO_2_-M delivered a high NH_3_ yield of 5.18 mg h^−1^ cm^−2^ at −1.7 V vs. Ag/AgCl and a Faraday efficiency of 88.05% at −1.4 V vs. Ag/AgCl in 0.1 M NaNO_3_ solution. It also maintained a Faraday efficiency of over 80% under five consecutive cycle tests. This work not only presents a highly promising electrode material, but also offers new insights into the rational design of Mo-based nanocomposites for NO_3_^−^RR.

## 2. Results and Discussion

### 2.1. Morphological and Structural Analysis of Catalysts

**Figure 1:**
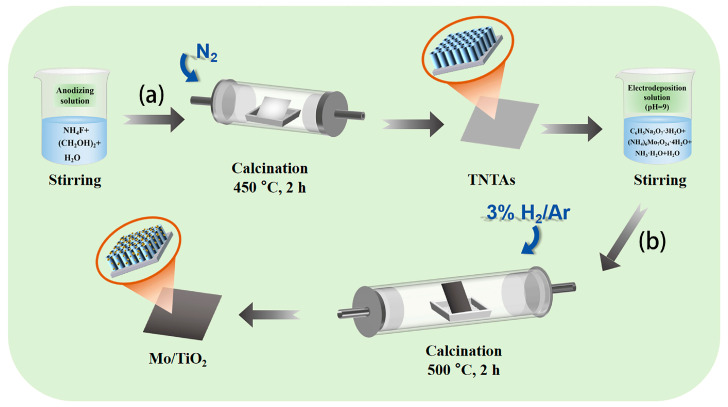
Schematic illustration of the two-step synthesis process of the Mo/TiO_2_ electrodes: (**a**) anodization and then calcination under a N_2_ atmosphere at 450 °C for 2 h to prepare TNTAs; (**b**) electrodeposition and then calcination in 3% H_2_/Ar at 500 °C for 2 h.

Figure 1 shows the two-step process to fabricate the Mo/TiO_2_ electrode. Firstly, TiO_2_ nanotube arrays were formed on Ti foil (TNTAs) by using anodization process (Figure 1a) [38], and then Mo was further loaded onto the TNTAs using the electrodeposition method and finally annealed under 3% H_2_/Ar atmosphere to prepare Mo/TiO_2_ (Figure 1b). The Mo/TiO_2_ can directly serve as the electrode for NO_3_^−^RR.

**Figure 2:**
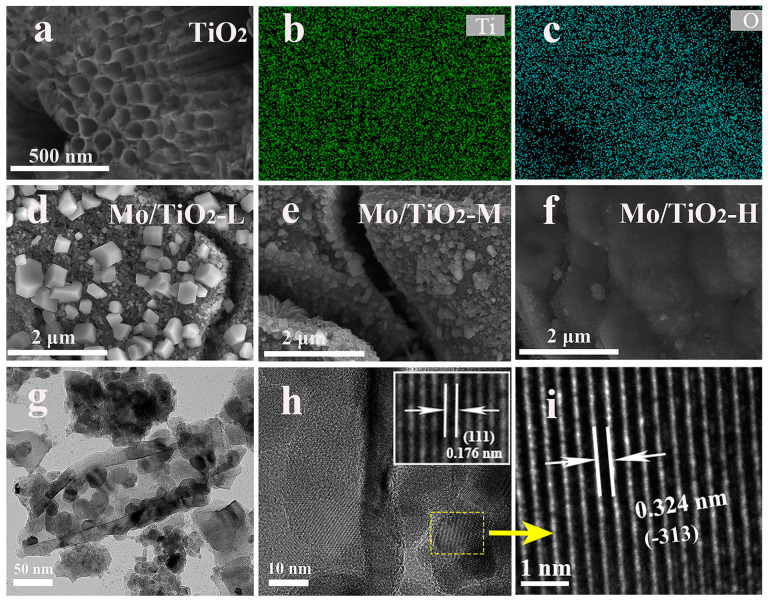
(**a**) SEM image of TNTAs, and (**b**,**c**) the corresponding EDS mapping images. SEM images of (**d**) Mo/TiO_2_-L, (**e**) Mo/TiO_2_-M, and (**f**) Mo/TiO_2_-H. (**g**,**h**) TEM and (**i**) HR-TEM images of Mo/TiO_2_-M.

As shown in Figure 2a, the nanotube structure of the TNTAs was observed, and TNTAs with a tube diameter of 100 nm uniformly covered the Ti foil. The EDS mapping images (Figure 2b,c) show that Ti and O elements are distributed on TNTAs. TNTAs with a nanotube array structure can be a promising substrate to support active materials, and can offer a further pathway for ion transfer from electrolyte to electrode [35]. It was observed that the particle size of molybdenum oxide particles grown on the surface of the nanotubes gradually decreased with the increase in molybdate concentration in the electrodeposition solution. The SEM image of Mo/TiO_2_-L (Figure 2d) displays some nanoparticles on TNTAs. With increased molybdate concentration, relatively small particles can be found on the surface of TNTAs, as shown in Figure 2e,f, which is ascribed to the kinetics of electrochemical deposition. In general, the concentration of the electrolyte is proportional to the uniformity of the electrodeposition. A high concentration of molybdate allows a sufficient amount of Mo species to be adsorbed onto the electrode surface, thus resulting in the uniform growth of the Mo layer. In the electrolyte with low molybdate concentration, the nucleation of Mo is controlled by the diffusion of Mo species. This is because there are insufficient Mo species near the electrode region, causing the Mo species to preferentially adsorb on the nucleated particles with a larger radius of curvature. Therefore, the particles with a larger size can be formed for Mo/TiO_2_-L (Figure 2d). To further confirm the nanocomposite structure, TEM measurement was carried out. The Mo/TiO_2_ active material was scraped off and dispersed in ethanol for TEM measurement. As shown in Figure 2g, the nanotube structure can be clearly observed with a wall thickness of 15 nm. Notably, some nanoparticles can be found inside and outside the nanotube with a size of 25–30 nm. The high-magnification TEM image (Figure 2h inset) shows a clear boundary between nanoparticles and nanotubes. A highly ordered fringe with an interplanar distance of 0.176 nm can be observed in the nanotube region (Figure 2h inset), which corresponds to the (111) plane of the TiO_2_ phase [39]. Moreover, the high-magnification TEM image of the nanoparticles (Figure 2i) displays a lattice fringe with a d-space of 0.324 nm, suggesting the (−313) plane of MoO_2_. The above results indicate the successful formation of a Mo/TiO_2_ nanocomposite structure by means of the electrochemical deposition process [40].

**Figure 3:**
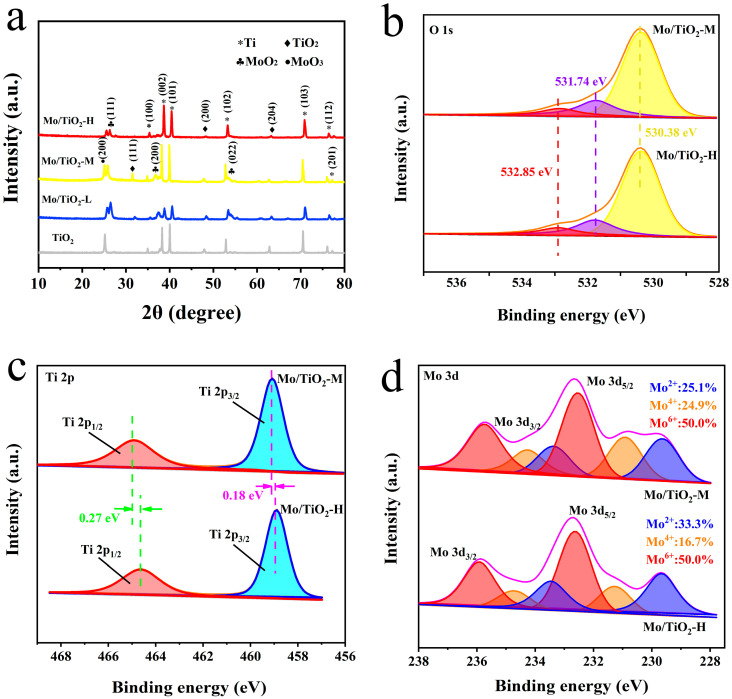
(**a**) XRD patterns of TiO_2_, Mo/TiO_2_-L, Mo/TiO_2_-M, and Mo/TiO_2_-H. High-resolution XPS spectra of Mo/TiO_2_-M and Mo/TiO_2_-H: (**b**) O 1s, (**c**) Ti 2p, and (**d**) Mo 3d regions.

The successful preparation of TNTAs and Mo/TiO_2_ electrodes could be further confirmed through XRD characterization. As shown in XRD patterns (Figure 3a), the peaks at 17.5°, 19.2°, 20.1°, 26.5°, 35.3°, 38.1°, and 38.7° (PDF#44-1294) are ascribed to the (100), (002), (101), (102), (103), (112), and (201) planes of Ti. The characteristic peaks at 15.7°, 20.0°, and 31.3° (PDF#97-001-5328) are observed in all samples, corresponding to the (111), (200), and (204) planes of TiO_2_. Moreover, the peaks at 12.9°, 18.50°, 26.7° (PDF#97-010-8875), and 12.5° (PDF#97-008-6426) correspond to the (111), (211), and (022) planes of MoO_2_ and the (200) plane of MoO_3_, respectively. The peaks of TiO_2_ can still be observed after Mo loading, indicating that the electrodeposition process cannot influence the crystal structure of TiO_2_. In addition, the intensity of new peaks corresponding to MoO_x_ decreased with the increase in molybdate concentration. This could be due to the smaller grain size of MoO_x_, which is ascribed to the SEM results. In addition, the grain size of samples was calculated using the Scherrer equation, D = Kγ/(Bcosθ), where K is Scherrer’s constant (0.89), γ is the wavelength of the X-rays (1.54056 Å), B is the half-peak height width, and θ is the Bragg angle. Therefore, the grain size of Mo/TiO_2_-L, Mo/TiO_2_-M, and Mo/TiO_2_-H was calculated to be 30.8, 26.8, and 22.6 nm, respectively.

The surface chemistry of Mo/TiO_2_-M and Mo/TiO_2_-H was further investigated using XPS. The survey scan XPS spectrum shows the photoelectron lines with binding energies (BEs) at three peaks of 532, 460, and 233 eV corresponding to the O 1s, Ti 2p, and Mo 3d signals (Figure S1). As shown in Figure 3b, the peaks of O 1s with BEs at 530.38 and 532.85 eV are attributed to lattice oxygen and physically adsorbed oxygen, respectively [41,42]. In addition, the peak with BE at 531.74 eV corresponds to chemically adsorbed oxygen. This could be due to the fact that the oxygen defects on the surface after H_2_ treatment, and the positively charged defection sites can adsorb O_2_ to become reactive oxygen species [34,43]. As displayed in Figure 3c, the peaks with BEs at 460.18 and 464.90 eV correspond to the spin-orbit splitting peak of Ti 2p_1/2_ and Ti 2p_3/2_, respectively, proving the existence of Ti^4+^ [44]. Notably, compared with the Mo/TiO_2_-H sample, the Ti 2p_1/2_ and Ti 2p_3/2_ peaks display positive shifts of 0.18 and 0.27 eV for Mo/TiO_2_-M, indicating that the structure of MoO_x_ could influence the local chemical states of Ti^4+^. According to a previous report, oxygen defect could be formed after H_2_ treatment, which can reduce Ti^4+^ into Ti^3+^ [45]. However, almost no Ti^3+^ was detected in the two samples, possibly due to the low amount of Ti^3+^. The positive shift could be explained by the grain size of MoO_x_, in which more interaction could occur between Mo and Ti atoms. Figure 3d demonstrates the Mo 3d XPS spectra of Mo/TiO_2_-M and Mo/TiO_2_-H. The Mo 3d in the samples are consist of three spin-orbit splitting components. The two peaks at BEs of 229.64 and 233.37 eV are attributed to the Mo 2d_5/2_ and Mo 2d_3/2_ of Mo^2+^ [46]. Additionally, the peaks at BEs of 230.91, 234.26, 232.54, and 235.74 eV are attributed to the Mo 2d_5/2_ and Mo 2d_3/2_ of Mo^4+^ and the Mo 2d_5/2_ and Mo 2d_3/2_ of Mo^6+^, respectively [46,47]. Notably, negative shifts in the peak position of Mo/TiO_2_-M can be found compared with Mo/TiO_2_-H. This indicates the electron transfer to Mo atoms. Therefore, the existence of electron transfer from Ti to Mo atoms is speculated. According to the d-band center theory proposed by Norskov et al. [48], the active site of Mo in Mo/TiO_2_-M could exhibit a rising d-band center compared with Mo/TiO_2_-H. This suggested that less electrons would fill the antibonding orbitals, thus increasing the adsorption energy between intermediate species and active site [49]. Hence, the binding energy of intermediate species in NO_3_^−^RR could be adjusted by rationally regulating the grain size of MoO_x_. Furthermore, the relative amounts of Mo in each valence state were calculated through the peak areas. As displayed in Figure 3d, it was found that Mo(IV) is more abundant in Mo/TiO_2_-M. This implies that the structure of MoO_x_ (Figure 2d–f and 3a) and the valence of Mo (Figure 3d) can be influenced by rationally designing the concentration of molybdate during electrodeposition.

### 2.2. Electrocatalytic Performance of Electrodes for Mo/TiO_2_

**Figure 4:**
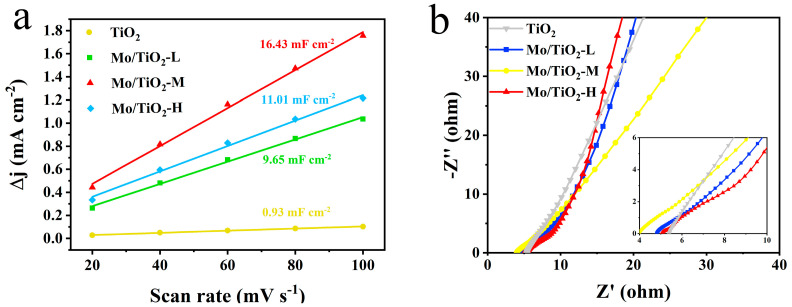
(**a**) Double-layer capacitances of TiO_2_, Mo/TiO_2_-L, Mo/TiO_2_-M, and Mo/TiO_2_-H electrodes. (**b**) Nyquist plots of TiO_2_, Mo/TiO_2_-L, Mo/TiO_2_-M, and Mo/TiO_2_-H electrodes.

CV, LSV, and EIS measurements were conducted in an H-type three-electrode system in 0.05 M Na_2_SO_4_ electrolyte to compare the electrochemical performance of different samples. The CV tests were firstly conducted at different scan rates (Figure S2) to determine the electric double-layer capacitances (C_dl_). Notably, the electrochemical active surface area (ECSA) was positively correlated with C_dl_ [50,51]. The C_dl_ for TiO_2_, Mo/TiO_2_-L, Mo/TiO_2_-M, and Mo/TiO_2_-H were 0.93, 9.65, 16.43, and 11.01 mF cm^−2^, respectively (Figure 4a), demonstrating that Mo/TiO_2_-M has a significantly greater ECSA than other concentrations. The greater ECSA for Mo/TiO_2_-M can be ascribed to its appropriate particle size. As shown in Figure 2d–f, the high concentration of molybdate results in the formation of a dense MoO_x_ layer on the surface of TNTAs, thus leading to the reduction of the surface area for the nanoarray electrode. Conversely, Mo/TiO_2_-L, prepared with a low molybdate concentration, exhibits a larger size of particles, which decreases the solid-liquid contact surface. Therefore, the result indicates that Mo/TiO_2_-M could behave better for NO_3_^−^RR. This result can also be proven by the EIS measurement. Figure 4b shows the Nyquist plots of TiO_2_, Mo/TiO_2_-L, Mo/TiO_2_-M, and Mo/TiO_2_-H. In the high frequency region, the charge transfer resistance (R_ct_) and the electrolyte contact resistance (R_e_) are reflected by the intercepts of the radius of the high frequency arc on the real axis and the Nyquist plots, respectively [52]. The R_ct_ of the Mo/TiO_2_-M electrode is much smaller than that of the other electrode, indicating faster charge transition [53]. Moreover, since TiO_2_ is a semiconductor, its conductivity is the worst, resulting in the smallest R_ct_. At low frequency, the perpendicularity of the lines of Mo/TiO_2_-L and Mo/TiO_2_-H are as similar as TiO_2_, indicating that their ion diffusion is close.

**Figure 5:**
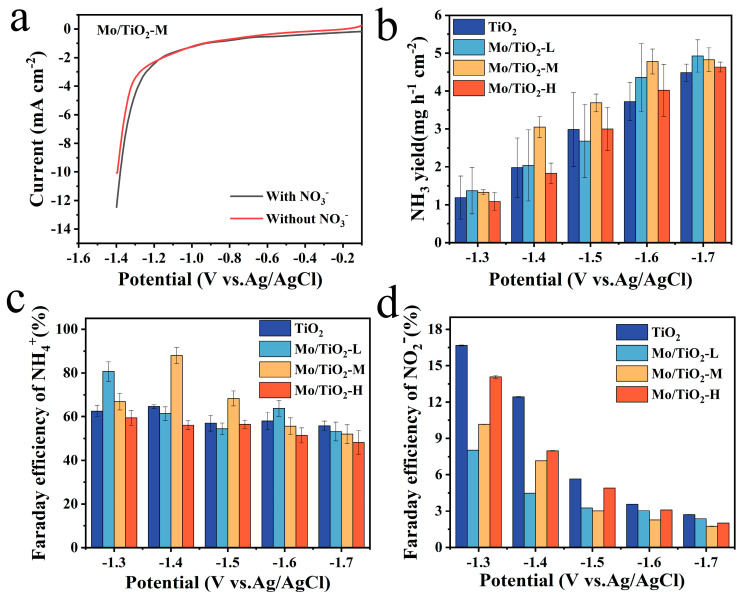
(**a**) LSV curves of Mo/TiO_2_-M in 0.05 M Na_2_SO_4_ solution with and without NO_3_^−^ at a scan rate of 5 mV s^−1^. The NH_3_ yield of (**b**) TiO_2_, Mo/TiO_2_-L, Mo/TiO_2_-M, and Mo/TiO_2_-H at the corresponding potentials. FE of NH_4_^+^ of (**c**) TiO_2_, Mo/TiO_2_-L, Mo/TiO_2_-M, and Mo/TiO_2_-H. FE of NO_2_^−^ of (**d**) TiO_2_, Mo/TiO_2_-L, Mo/TiO_2_-M, and Mo/TiO_2_-H.

The LSV curves of the Mo/TiO_2_-M catalyst were tested in the electrolyte with and without nitrate-N at a scan rate of 5 mV s^−1^ to characterize whether it has NO_3_^−^RR catalytic properties. As shown in Figure 5a, it is evident that the current density of the LSV curve with NO_3_^−^ in 0.05 M Na_2_SO_4_ is larger than that of the other one, ranging from −1.1 V vs. Ag/AgCl to −1.4 V vs. Ag/AgCl, which proves that Mo/TiO_2_-M has NO_3_^−^RR properties. Moreover, it is widely known that nitrate reduction is an eight-electron transfer process. In addition, the NH_3_ yield and Faraday efficiency of samples are essential factors in evaluating NO_3_^−^RR electrocatalytic properties. For this reason, UV spectroscopy was used to measure the concentration of NH_3_^+^ and NO_2_^−^. As shown in Figure S3, the linear fitting results correspond to the absorbance versus concentration curves of NH_3_^+^ and NO_2_^−^. The concentrations of the corresponding ions can be obtained from the measured absorbance and the standard curve.

Figure 5b shows that the most preferred NH_3_ production of TiO_2_, Mo/TiO_2_-L, Mo/TiO_2_-M, and Mo/TiO_2_-H was reached at −1.6 V vs. Ag/AgCl, as the NH_3_ yield did not change much as the voltage continued to increase. Additionally, the highest NH_3_ productions of samples are reached at −1.7 V vs. Ag/AgCl. Among them, Mo/TiO_2_-M and Mo/TiO_2_-H exhibit the highest NH_3_ yields, around 5.18 mg h^−1^ cm^−2^ and 5.20 mg h^−1^ cm^−2^, respectively_._ Moreover, as shown in Figure 5c, the highest FE was 88.05%, corresponding to Mo/TiO_2_-M at −1.4 V vs. Ag/AgCl. Meanwhile, the highest FEs of 65.50%, 85.98%, and 63.91% were achieved for TiO_2_, Mo/TiO_2_-L, and Mo/TiO_2_-H, respectively. It can be seen that the FEs of Mo/TiO_2_-M remain at a high value at different voltages. This indicates that Mo/TiO_2_-M has superior NO_3_^−^RR performance, which may be due to the appropriate grain size of MoO_x_ in Mo/TiO_2_-M. Moreover, Table S1 compares the NO_3_^−^RR performance of Mo/TiO_2_-M with other previously reported electrodes. The FE and NH_3_ yields of the Mo/TiO_2_-M electrode are comparable to most of the previous cathodes, further indicating the good activity of the as-prepared Mo/TiO_2_-M electrode [27,54,55,56,57,58,59].

Furthermore, the generation properties of the byproduct NO_2_^−^ at each potential were also evaluated, as shown in Figure 5d. It was found that all MoO_x_-loaded samples inhibited NO_2_^−^ generation compared to TiO_2_, with the strongest inhibition achieved by Mo/TiO_2_-M at voltages of −1.5–1.7 V vs. Ag/AgCl. Additionally, the change in the amount of NO_3_^−^ in the electrolyte (Figure S5a) was measured, and then the amount of N_2_ produced during the NO_3_^−^RR reaction was calculated. As shown in Figure S5b, the quantity of N_2_ decreases with the increase in voltage, and no N_2_ is produced at −1.7 V vs. Ag/AgCl for any of the electrodes.

**Figure 6:**
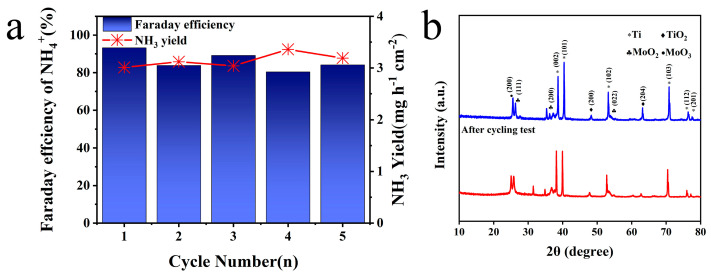
(**a**) NH_3_ yields and FE of Mo/TiO_2_-M at −1.4 V (vs. RHE) for five cycling tests. (**b**) XRD patterns of Mo/TiO_2_-M before and after cycling tests.

Based on the above test and analysis, Mo/TiO_2_-M was selected to operate a cycling test at −1.4 V vs. Ag/AgCl. As shown in Figure 6a, NH_3_ production exceeded 3 mg h^−1^ cm^−2^ and that Faraday efficiency stabilized over 80% in all groups. Cycling evaluation further highlighted Mo/TiO_2_-M’s outstanding and steady NO_3_^−^RR performance at −1.4 V vs. Ag/AgCl. Furthermore, a leaching test was conducted to determine possible Mo species in the electrolyte [60]. After the NO_3_^−^RR process, the concentration of Mo elements in the electrolyte was measured at only 0.0148 mg/L, suggesting almost no dissolution of Mo. Figure S6 displays the SEM image of the Mo/TiO_2_-M electrode after cycling tests. It is clear that no significant change can be observed, and nanoparticles are evident on the surface of the electrode. Figure 6b compares the XRD patterns of the Mo/TiO_2_-M electrode before and after cycling tests. Notably, almost no change can be seen, indicating the good structure stability of the Mo/TiO_2_-M electrode.

## 3. Experimental Methods

### 3.1. Materials

Titanium foil (0.3 mm thickness, 99.9% of purity) was supplied from Tianjin Xingboguangwang Metal Co (Tianjin, China). Ammonium molybdate tetrahydrate ((NH_4_)_6_Mo_7_O_24_·4H_2_O), sodium nitroferricyanide dihydrate (C_5_FeN_6_Na_2_O·2H_2_O), and sodium sulfate (Na_2_SO_4_) were purchased from Shanghai Titan Scientific Co. (Shanghai, China). Chemical reagents such as sodium citrate trihydrate (C_6_H_5_Na_3_O_7_·3H_2_O), salicylic acid (C_7_H_6_O_3_), ammonia (NH_3_·H_2_O), ammonium fluoride (NH_4_F), potassium nitrate (KNO_3_), sodium hydroxide (NaOH), sulfamic acid (NH_2_SO_3_H), hydrochloric acid (HCl, 36–38%), sulfanilamide (C_6_H_8_N_2_O_2_S), sodium hypochlorite (NaClO), phosphoric acid (H_3_PO_4_), ethylene glycol (CH_2_OH)_2_, anhydrous ethanol (C_2_H_5_OH), and naphthylenediamine hydrochloride (C_12_H_14_N_2_·2HCl) were obtained from Kelong Chemical Co (Chengdu, China). All chemical reagents used for the synthesis of Mo/TiO_2_ were of analytical grade and were used as-is. Deionized water (18.25 MΩ·cm) used throughout the experiment was from an ultrapure water system.

### 3.2. Preparation of Electrode Material

#### 3.2.1. Pre-Treatment of Ti

The pre-cut Ti foil with 2 cm × 1 cm × 0.03 cm dimensions was sanded with 800- and 1200-mesh metallographic sandpaper to produce a silvery luster on the surface. Subsequently, the processed Ti sheets were ultrasonically washed with ethanol for 20 min and deionized water for 20 min [61]. Then, the Ti foil was rinsed with deionized water to effectively remove the residual organic impurities on the surface during ultrasonic cleaning.

#### 3.2.2. Preparation of TNTAs

Uniformly aligned TNTAs were grown on the surface of Ti foil using an anodic oxidation strategy. Dissolve ammonium fluoride (NH_4_F) (0.5 g) in deionized water (2 mL) and ethylene glycol (C_2_H_6_O_2_) (98 mL) to prepare electrolyte solution successively. Then, the Ti foil and platinum (Pt) electrode, which were cleaned as described above, were used as anode and cathode, respectively. The distance between the anode and the cathode was adjusted to be 3 cm approximately and reacted by a constant voltage device at 30 V for two hours. After that, the Ti foil was washed with anhydrous ethanol and placed in a tube furnace to be calcined for 2 h at 450 °C in an air environment. Finally, TNTAs with anatase phase were successfully produced.

#### 3.2.3. Preparation of Mo/TiO_2_ Electrode

A certain mass of ammonium molybdate tetrahydrate solid ((NH_4_)_6_Mo_7_O_24_·4H_2_O) and 2.2075 g of sodium citrate (C_6_O_7_H_5_Na_3_·2H_2_O) were added into deionized water (50 mL) to prepare four groups of samples (0 M, 0.05 M, 0.1 M, and 0.2 M molybdate). NH_3_·H_2_O was added to the mixture after stirring to bring the pH up to 9. The prepared TNTA was used as the working electrode, a platinum (Pt) sheet as the auxiliary electrode, and Ag/AgCl as the reference electrode. Meanwhile, the current density was −20 mA cm^−2^, and the electrodeposition time was set to 20 min. After the process was completed, the samples were cleaned with deionized water and then put in a tube furnace to be calcined in 3% H_2_/Ar at the rate of 50 mL min^−1^ for 2 h. Finally, the electrodes were successfully produced, and the materials with different concentration of molybdate (0, 0.05 M, 0.1 M, and 0.2 M) were named as TiO_2_, Mo/TiO_2_-L, Mo/TiO_2_-M, and Mo/TiO_2_-H, respectively.

### 3.3. Characterization

The Ag/AgCl potential was converted to a reversible hydrogen electrode (RHE) using the Nernst equation: $E_{RHE}=E_{Ag/AgCl}+0.059\times pH+0.197$. All data were collected on a CHI660E electrochemical workstation (Shanghai CH Instruments, Shanghai, China). The crystal structure of the processed samples was characterized through X-ray diffraction (XRD) using a MiniFlex600 (Rigaku, Tokyo, Japan) with Cu-Kα radiation (λ = 0.154056 nm) at an ambient temperature (25 °C) and 2θ values ranging from 10 to 80 °. Energy dispersive spectroscopy (EDS) spectra and scanning electron microscopy (SEM) graphics were obtained using an FEI Quanta 250 (Regulus 8230U, Hitachi, Japan). A K-alpha spectrometer (Thermo Scientific, Waltham, MA, USA) equipped with a monochromatic Al Kα X-ray source (1486.6 eV photons) was used to perform X-ray photoelectron spectroscopy (XPS). Images from a transmission electron microscope (TEM) were captured at 200 kV using a Libra 200FE (Zeiss, Oberkochen, Germany). The concentration of ions in electrolyte was determined using an optima 7000DV inductively coupled plasma optical emission spectrometer (ICP-OES, Thermo Scientific, Waltham, MA, USA)

### 3.4. Electrochemical Measurement

The above-made electrode was used as the working electrode (1 cm × 1 cm), a Pt sheet as the counter electrode (1 cm × 1 cm), and Ag/AgCl as the reference electrode, placed in an H-type electrolyzer. The electrolyte was 0.1 M NO_3_^−^-N solution containing 0.05 M Na_2_SO_4_. The nitrate solution was then tested by i-t for one hour, testing the NH_3_ production at constant voltage to obtain the optimum operating voltage. The reacted cathode solution was collected for subsequent measurements.

Cyclic voltammetry (CV) was performed at −0.1 to 0 V against Ag/AgCl with a sampling rate of 20 to 100 mV s^−1^ with an interval of 20 mV s^−1^ in order to estimate the double layer capacitance (Cdl) of samples. Electrochemical impedance spectroscopy (EIS) was performed in an aqueous solution comprising 0.05 M Na_2_SO_4_. Additionally, the EIS measurements were performed in a frequency range from 0.01 to 100,000 Hz with an amplitude of sinusoidal AC voltage of 5 mV and 2 points per decade. Then, linear scanning voltammetry (LSV) was performed in the voltage range of −1.4–0 V (vs. RHE) for the tests.

### 3.5. Determination of Ion Concentration

UV spectroscopy was used to measure the concentrations of ammonium and nitrite ions. The method is as follows:

#### 3.5.1. Nitrite-N Detection

Five groups of sodium nitrite solutions (0, 0.25, 0.5, 1, 2, and 3 μg/mL) were prepared separately, 1 mL of the Griess reagent was added, and then the solution was left to develop color for 10 min. The absorbance was measured at 540 nm using a UV spectrophotometer, and the standard concentration curve of nitrite was plotted. Diluting the cathode solution after the reaction to a measurable concentration range, 1 mL of the Griess reagent was added into it, and then the absorbance was measured. The corresponding nitrite concentration was calculated according to its standard concentration graph.

#### 3.5.2. NH_3_-N Detection

Five groups of NH_4_^+^ solution (0, 1, 2, 3, and 4 μg/mL, respectively) were prepared. A total of 2 mL of colorant, 1 mL of oxidant, and 200 μL of catalyst were added sequentially, and then the solution was left to develop color by avoiding light for one hour. The absorbance was measured at 660 nm using a UV spectrophotometer, and the standard concentration curve of NH_4_^+^ was plotted. Diluting the NH_4_^+^ concentration after the reaction to a measurable concentration range, the three solutions were added in turn like the steps mentioned above, and then the absorbance was measured. The corresponding NH_4_^+^ concentration was calculated according to its standard concentration graph.

### 3.6. Product Calculation (Yield and Faraday Efficiency)

The NH_3_ yield was calculated using the following equation:

$$Yield=\frac{C({NH}_{3})\times V}{S\times t}$$

where C (NH_3_) is the measured concentration of NH_3_−N (aq), V (50 mL) is the volume of the cathode cell electrolyte, t (3600 s) is the electrochemical reaction time, and S (1 cm × 1 cm) is the surface area of the working electrode.

The Faraday efficiency (FE) was calculated using the following equation:

$$FE=\frac{3\times F\times C({NH}_{3})\times V}{17\times Q}\times 100\%$$

where F is Faraday’s constant (96485 C mol^−1^) and Q is the total charge across the electrolyte.

## 4. Conclusions

In summary, Mo/TiO_2_ nanocomposite material was fabricated through a two-step method. Such Mo/TiO_2_ can be used as a catalyst for NO_3_^−^RR and have good catalytic activity and performance. Both SEM and TEM results illustrated that MoO_x_ nanoparticles were uniformly loaded onto TNTAs. It was observed that the particle size of molybdenum oxide particles grown on the surface of the nanotubes can be controlled by regulating the molybdate concentration in the electrodeposition process. In addition, the XPS results revealed the existence of electron transfer from Ti to Mo atoms in the Mo/TiO_2_ nanocomposite material, which could be controlled by regulating the grain size of MoO_x_. The electron transfer facilitated the upward shift of the d-band center of the active Mo site, thus increasing the adsorption energy between intermediate species and active site in the NO_3_^−^RR process. Furthermore, the optimized Mo/TiO_2_-M electrode with more Mo(IV) displayed good activity and stable performance for NO_3_^−^RR. It delivered a Faraday efficiency of 88.05% and a NH_3_ yield of 3.0 mg h^−1^ cm^−2^ at −1.4 V vs. Ag/AgCl. Therefore, Mo/TiO_2_-M was experimentally proven to be an attractive electrode material for NO_3_^−^RR with excellent performance.

## Data Availability

Data are contained within the article.

## Supplementary Materials

The following supporting information can be downloaded at: https://www.mdpi.com/article/10.3390/molecules29122782/s1, Figure S1: XPS full spectrum map of Mo/TiO_2_-M and Mo/TiO_2_-H; Figure S2: CV curves at a scan rate of 20–100 mV s^−1^ for (a) TiO_2_, (b) Mo/TiO_2_-L, (c) Mo/TiO_2_-M, and (d) Mo/TiO_2_-H.; Figure S3: Standard curves for ion concentration of (a) NH_4_^+^ and (b) NO_2_^−^; Figure S4: LSV curves for (a) four samples, (b) TiO_2_, (c) Mo/TiO_2_-L, and (d) Mo/TiO_2_-H in 0.05 M Na_2_SO_4_ solution with and without NO_3_^−^ at a scan rate of 5 mV s^−1^. Figure S5. Quantity of N_2_ of TiO_2_, Mo/TiO_2_-L, Mo/TiO_2_-M, and Mo/TiO_2_-H at the corresponding potentials. Figure S6. SEM image of Mo/TiO_2_-M after five cycling tests. Table S1. Comparison of the NO_3_^−^RR performance for Mo/TiO_2_ with other electrocatalysts.
