# Supplementary material for: Molybdenum-Modified Titanium Dioxide Nanotube Arrays as an Efficient Electrode for the Electroreduction of Nitrate to Ammonia
Source: Molecules. 2024 Jun 11;29(12):2782. doi: 10.3390/molecules29122782 (PMC11206489; doi:10.3390/molecules29122782)
Supplement: Supplementary file 1 [file molecules-29-02782-s001.zip › molecules-3030999-supplementary.pdf]

Electronic Supplementary Information

Molybdenum-Modified Titanium Dioxide Nanotube Arrays as an Efficient Electrode for the Electroreduction of Nitrate to Ammonia

Huixi Chen, Wenqi Hu, Tingting Ma, Yixuan Pu, Senhao Wang, Yuan Wang,*
Shaojun Yuan*

Low-carbon Technology & Chemical Reaction Engineering Lab, College of Chemical Engineering, Sichuan University, Chengdu 610065, P. R. China
*Corresponding author: wangyuan2022@scu.edu.cn (Y.W.); ysj@scu.edu.cn (S.Y.);
Tel/Fax: +86-28-85405201

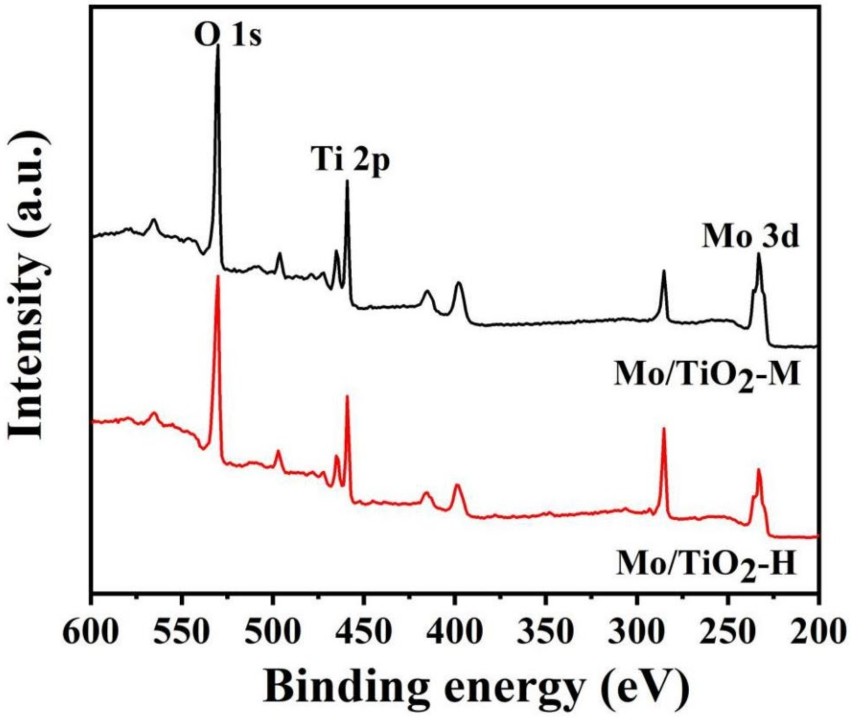

Figure S1. XPS full spectrum map of Mo/TiO_2-M and Mo/TiO_2-H.

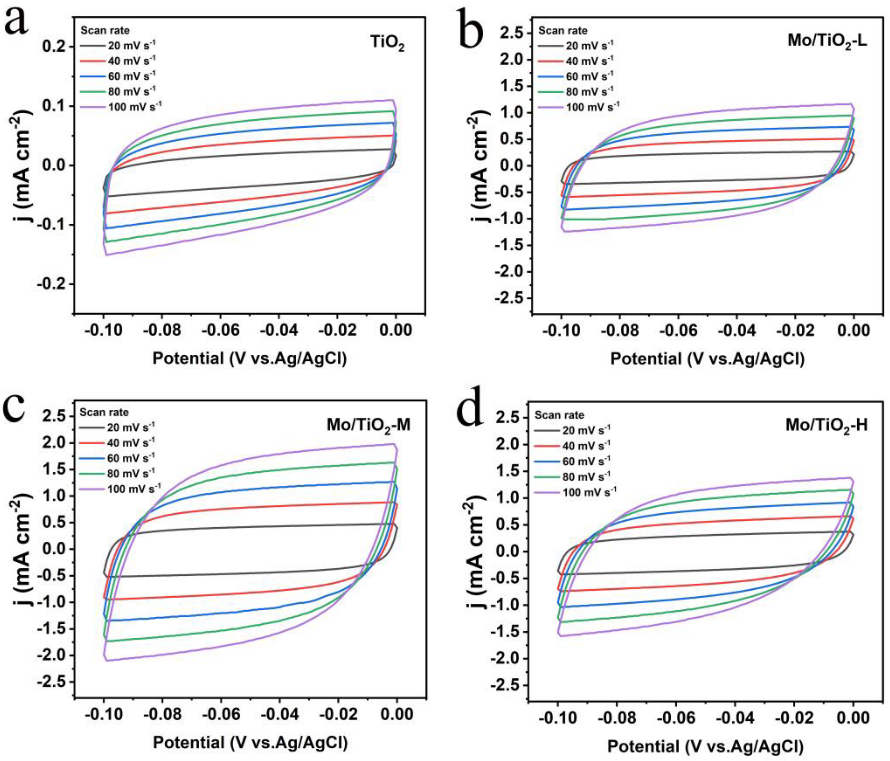

Figure S2. CV curves at scan rate of 20–100 mV s^{-1} for (a) TiO_2, (b) Mo/TiO_2-L, (c) Mo/TiO_2-M and (d) Mo/TiO_2-H.

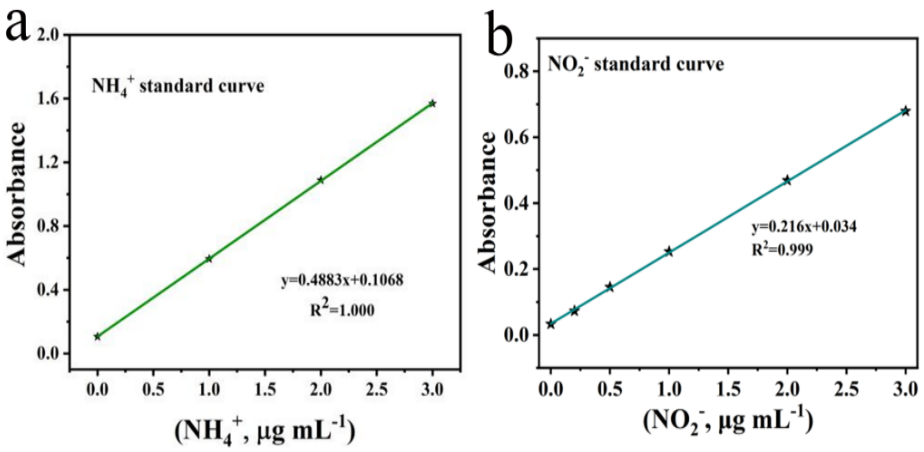

Figure S3. Standard curves for ion concentration of (a) NH_4^+ and (b) NO_2^-.

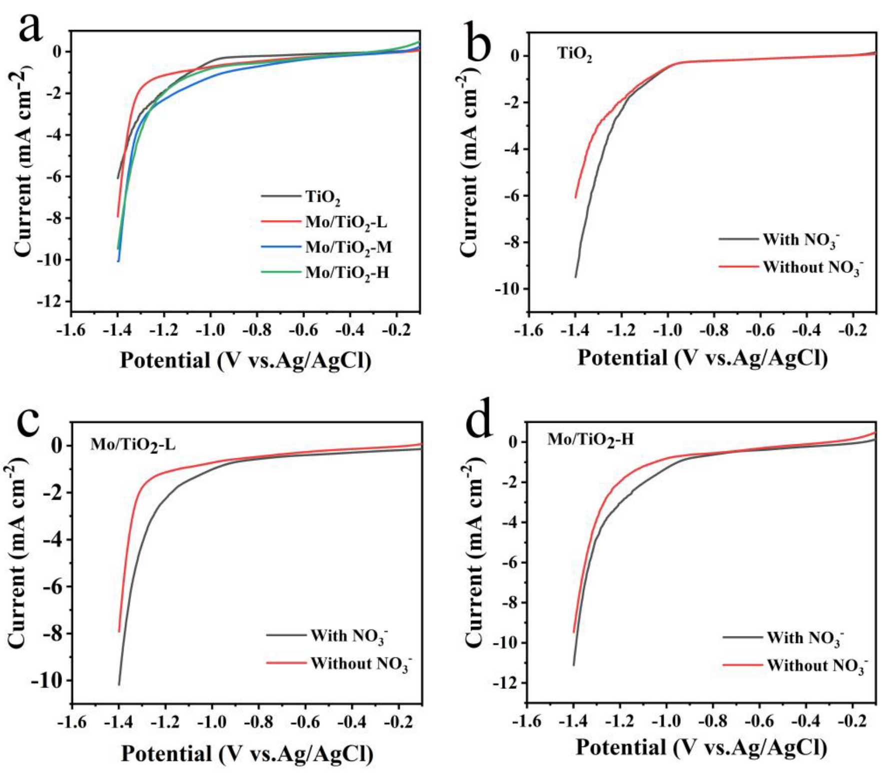

Figure S4. LSV curves for (a) four samples, (b) TiO_2, (c) Mo/TiO_2-L, and (d) Mo/TiO_2-H in 0.05 M Na_2SO_4 solution with and without NO_3^- at a scan rate of 5 mV s^{-1}.

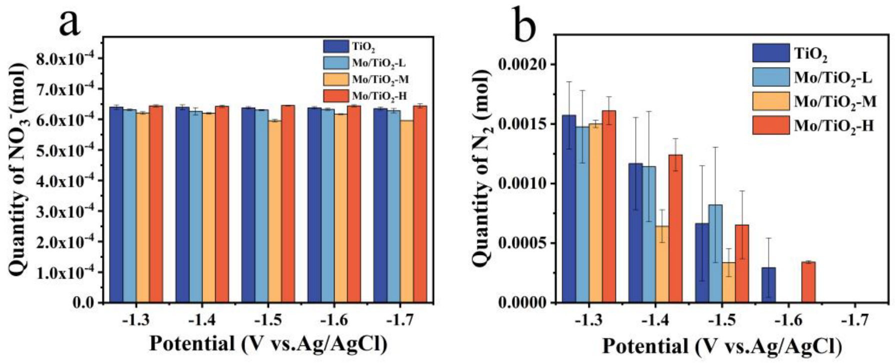

Figure S5. Quantity of N_2 of TiO_2, Mo/TiO_2-L, Mo/TiO_2-M and Mo/TiO_2-H at the corresponding potentials.

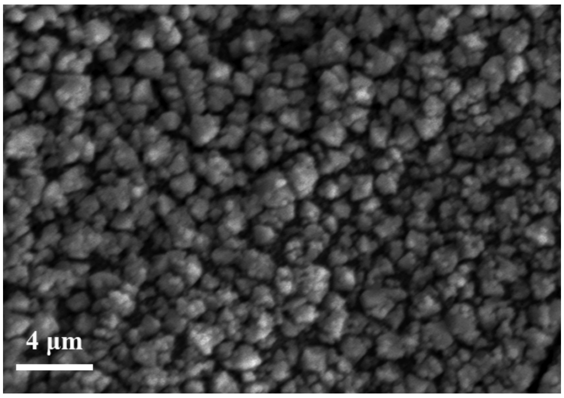

Figure S6. SEM image of Mo/TiO_2-M after five cycling tests.

Table S1. Comparison of the NO_3^-RR performance for Mo/TiO_2 with other electocatalysts.

Electrodes	electrolyte	potential（V vs.RHE）	FE (%)	NH_3 yield	Ref
Mo/TiO_2	0.1 M KNO_3	-1.4	88.05	3.0 mg h^{-1} cm^{-2} cat	This work
CuO NWAs@Co	0.006 M KNO_3	-0.85	85.9	0.436 mg h^{-1} cm^{-2} cat	Nature Energy. 2020, 5, 605–613
Fe1/NC-900	0.5 M KNO_3	-0.9	86	18.8 mg h^{-1} cm^{-2} cat	Appl. Catal., B. 2023, 323, 122181
Pd/BCN	0.2 M KNO_3	-1	91.79	12.71 mg h^{-1} cm^{-2} cat	J. Colloid Interface Sci. 2024, 664, 84–95
Ag1/NOCNT	1 M KNO_3	-1	97.9	90 mol h^{-1} g_{Ag}^{-1}	Appl. Catal., B. 2023, 331, 122687
MoO_2/MP	0.1 M $NaNO_3$	-0.8	94.5	8.678 mg h^{-1} cm^{-2} cat	Colloids Surf., A. 2023, 657, 130549
Rh@Cu	0.1 M KNO_3	-0.2	93	1.27 mmol h^{-1} cm^{-2}	Angew. Chem. Int. Ed. 2022, 61, 23
$Cu_{50}Ni_{50}$	100 mM KNO_3	0.25	99	/	J. Am. Chem. Soc. 2020, 142, 5702–5708
